# PEPMatch: a tool to identify short peptide sequence matches in large sets of proteins

**DOI:** 10.1186/s12859-023-05606-4

**Published:** 2023-12-18

**Authors:** Daniel Marrama, William D. Chronister, Luise Westernberg, Randi Vita, Zeynep Koşaloğlu-Yalçın, Alessandro Sette, Morten Nielsen, Jason A. Greenbaum, Bjoern Peters

**Affiliations:** 1 Division of Vaccine Discovery, La Jolla Institute for Immunology, La Jolla, San Diego, CA USA; 2 University of California San Diego School of Medicine, La Jolla, San Diego, CA USA; 3 Department of Health Technology, Technical University of Denmark, Lyngby, Denmark

**Keywords:** Peptide matching, T-cell epitopes, Sequence searching, K-mer mapping, BLAST comparison, Benchmarking, Immunology

## Abstract

**Background:**

Numerous tools exist for biological sequence comparisons and search. One case of particular interest for immunologists is finding matches for linear peptide T cell epitopes, typically between 8 and 15 residues in length, in a large set of protein sequences. Both to find exact matches or matches that account for residue substitutions. The utility of such tools is critical in applications ranging from identifying conservation across viral epitopes, identifying putative epitope targets for allergens, and finding matches for cancer-associated neoepitopes to examine the role of tolerance in tumor recognition.

**Results:**

We defined a set of benchmarks that reflect the different practical applications of short peptide sequence matching. We evaluated a suite of existing methods for speed and recall and developed a new tool, PEPMatch. The tool uses a deterministic *k*-mer mapping algorithm that preprocesses proteomes before searching, achieving a 50-fold increase in speed over methods such as the Basic Local Alignment Search Tool (BLAST) without compromising recall. PEPMatch’s code and benchmark datasets are publicly available.

**Conclusions:**

PEPMatch offers significant speed and recall advantages for peptide sequence matching. While it is of immediate utility for immunologists, the developed benchmarking framework also provides a standard against which future tools can be evaluated for improvements. The tool is available at https://nextgen-tools.iedb.org, and the source code can be found at https://github.com/IEDB/PEPMatch.

**Supplementary Information:**

The online version contains supplementary material available at 10.1186/s12859-023-05606-4.

## Background

Tools to compare nucleotide or amino-acid sequences, such as BLAST, are some of the most used bioinformatic methods [1]. Performing sequence alignments can lead to functional and evolutionary insights at the level of whole genes and proteins. Tools such as MUSCLE [2], DIAMOND [3], and MMSeqs2 [4] were created to speed up the alignment process beyond BLAST, increase recall, and address specific challenges in alignments.

Immunology researchers who study T-cell epitopes often apply general-purpose alignment tools. Such epitopes are typically linear peptides bound to major histocompatibility complex (MHC) molecules that are presented on the surface of host cells. These complexes enable T cells of the immune system to test them for binding to their T cell receptors. For MHC Class I, epitopes are typically 8–11 residues in length [5]. For MHC class II, epitopes are typically 13–17 residues in length [6], though shorter and longer peptides can be bound. A common research question is whether MHC-presented peptides are found within the proteins expressed by a given organism (its proteome), such as a pathogen, an allergen, or the host itself. We have compiled four real-life use cases from our own work as examples of the types of questions typically asked.

1. The Immune Epitope Database (IEDB) has curators combing the scientific literature to catalog epitopes and the experiments characterizing them [7]. If there is no literature information on the specific protein that an epitope is derived from, such source proteins are assigned by searching proteomes for exact matches.
2. The emergence of the novel coronavirus (SARS-CoV-2) in late 2019 led to an ongoing pandemic, causing global health, social, and economic disturbance. Researchers attempted to understand the nature of this virus, including exploring the possibility of immune cross-reactivity with other endemic viruses. Peptides from SARS-CoV-2 were found to share similarities with peptides from the four of the most common human coronaviruses (HCoV-229E, HCoV-HKU1, HCoV-NL63, and HCoV-OC43) [8], using a similarity metric based on the number of mismatches between peptides.
3. T lymphocytes of the immune system can recognize cancer cells expressing mutated proteins through their presentation of “neoepitopes.” Such neoepitopes have amino acid substitutions compared to their unmutated sequence, allowing them to be recognized as non-self. Comparing neoepitope sequences against the host-proteome can aid in determining if the same sequence is found elsewhere and might thus be tolerated.
4. Cow’s milk allergy is the most common pediatric food allergy, affecting nearly 2% of all children in the United States [9]. Reactions to cow milk allergens can be severe and makeup 8–15% of fatal or near-fatal food-induced anaphylaxis [10]. Researchers have postulated that the conservation of cow’s milk peptides in the human host may affect their allergenicity, with the less conserved peptides being more likely to cause allergic responses. In a recently published study [11], we examined a set of Cow’s milk peptides screened for allergic responses and found the best match in the human proteome for each. Of the peptides conserved at 100% homology, every single one was non-reactive, and the majority of the reactive peptides were poorly conserved in comparison.

As the examples above show, the matches of interest are identical peptides or allow for minimal mismatches. The currently available tools, such as BLAST, were not created for this particular task. They were developed and optimized to align longer sequences and lower sequence similarity. While they allow users to set parameters to tweak the methods for shorter sequence matches, such as the epitope use cases we describe above, our work demonstrates that they are not ideal and are not guaranteed to find every possible match.

We used several common sequence alignment tools and real data from the applications mentioned above as tests to establish benchmarks for speed and recall. We also developed our own tool, PEPMatch, which is publicly available and is hosted on the Immune Epitope Database (https://nextgen-tools.iedb.org). PEPMatch uses a non-alignment, deterministic *k*-mer mapping algorithm, which first preprocesses the proteome desired to search against and achieves a significant search speed increase compared with the other sequence alignment tools while still guaranteeing high recall. We have published the code used to benchmark these tools to facilitate collaborations with external users who may be incentivized to improve performance with better tools.

## Implementation

### Collection of relevant tools and algorithms

Four string searching algorithms and five additional tools, including the newly developed PEPMatch tool, were used to find epitope matches against a reference proteome. The four string searching algorithms are also deterministic; they can only find exact matches, and thus, they are only tested on the first dataset. The names of these algorithms are Boyer-Moore, Horspool, Knuth-Morris-Pratt (KMP), and the Z algorithm [13–16]. These algorithms were re-implemented in Python version 3.9 using previously published literature as references. We used the five other tools that could perform both exact matching and finding matches with substitutions as standalone binaries with Python version 3.9 wrappers written to standardize metrics for benchmarking. We downloaded the BLAST bin files from the NCBI website, version 2.10.0. The Biopython library, version 1.78, was used to run the BLAST algorithm locally. To allow maximum capture of true positives, we set the E-value threshold to 100 for exact match searching and 10,000 for mismatch searching. Both the DIAMOND and MMseqs2 tools have downloadable standalone binaries hosted on GitHub that were used for benchmarking, and their parameters were also set to allow for the capture of true positives for short sequences. For DIAMOND, the E-value was set to 10,000, k (the number of alignments to report per query) to 100, and the “ultra-sensitive” flag was passed. Lastly, for Mmseqs2, we used an E-value of 10,000 and set the tool’s sensitivity to 7, which is the highest possible. Another tool, NmerMatch (unpublished; https://github.com/IEDB/NmerMatch), is a peptide-searching tool written in the Perl programming language. A Linux machine was used to run the benchmarking code with a 16-core Intel i9-9900 K CPU @ 3.60 GHz, 32 GiB of RAM, and a Samsung 970 EVO Plus 1 TB SSD.

### Application datasets

We compiled four separate datasets to test these tools. There were three main peptide searching objectives associated with these datasets: finding exact matches, finding matches with mismatches (residue substitutions), and finding the best match (match with the least substitutions). Each of these datasets represents a unique application within immunology research. All method implementations, Python wrappers for benchmarking, and datasets are available within the GitHub codebase and the benchmarking framework at github.com/IEDB/PEPMatch.

#### Exact matching: MHC class I eluted ligands dataset

To compare the performance of the tools for exact match epitope searching within a reference proteome, we randomly selected 1000 9-mer HLA ligands from a recent paper [12], and we also shuffled these 1000 peptides into random sequences (Additional file 1: Table S1) to make sure the methods don’t incorrectly match these. We downloaded the human reference proteome from UniProt to search these epitopes within (UniProt Proteome ID: UP000005640). The four string searching algorithms can only be benchmarked for this dataset as they only perform exact matching.

#### Mismatching: SARS-CoV-2 and neoepitope datasets

To test the ability of these tools and algorithms to find peptides with limited mismatches (amino acid substitutions), we used two separate datasets with applications in infectious disease research and cancer research. A dataset containing 628 SARS-CoV-2 peptides (Additional file 2: Table S2) of varying lengths [8] was used to search against the entirety of the betacoronavirus genus proteins found in the NCBI RefSeq database [17]. We searched these peptides against this enormous set of proteins for up to and including two mismatches. Next, we used a dataset of 620 neoepitopes, all 15-mers (Additional file 3: Table S3), taken from the Cancer Epitope Database and Analysis Resource (CEDAR) [18], a freely accessible resource for cancer epitopes, to search against the human reference proteome taken from UniProt (UniProt Proteome ID: UP000005640). We searched these neoepitopes for up to and including three mismatches.

#### Best match: milk allergen dataset

The best match is defined as the peptide within the given proteome with the least number of amino acid substitutions. We used 677 15-mers (Additional file 4: Table S4) from a recent study [11] derived from cow milk proteins and screened across donors who had severe milk allergies. All of these peptides were searched in the human proteome (UniProt Proteome ID: UP000005640) to find the best match, the same way that was done in the study.

### PEPMatch: *k*-mer mapping algorithm

We developed PEPMatch to ensure all short peptide matches at a given mismatch frequency would be found. Our algorithm is based on matching shorter *k*-mers by breaking up the given proteome into all possible *k*-mers. We store all *k*-mers mapped to their index positions within the proteome as a key-value database. This preprocessing step is performed only once per proteome and per given *k* value. The hash table data structure allows for extremely fast lookup times when the preprocessed data is read into memory. Both exact matching of a peptide and searching for a peptide with mismatches can capitalize on hash table lookups and are explained separately below.

We store the *k*-mer to index mapping in a SQLite database or a serialized pickle format, depending on the matching task, either exact matching or matching with mismatches. The peptides queried for searching are passed and broken up into *k*-mers of the same size *k* as the preprocessed proteome. They can be broken up as *k*-mers using a rolling window of one amino acid. Alternatively, the peptides can be generated such that the total coverage of the peptide is obtained using the least number of *k*-mers, even if there is some overlap with the last two *k*-mers. Lookups are performed, and depending on the type of search (exact matching versus mismatching), specific calculations are executed to find matches; this is explained below.

#### Exact matching

**Fig. 1:**
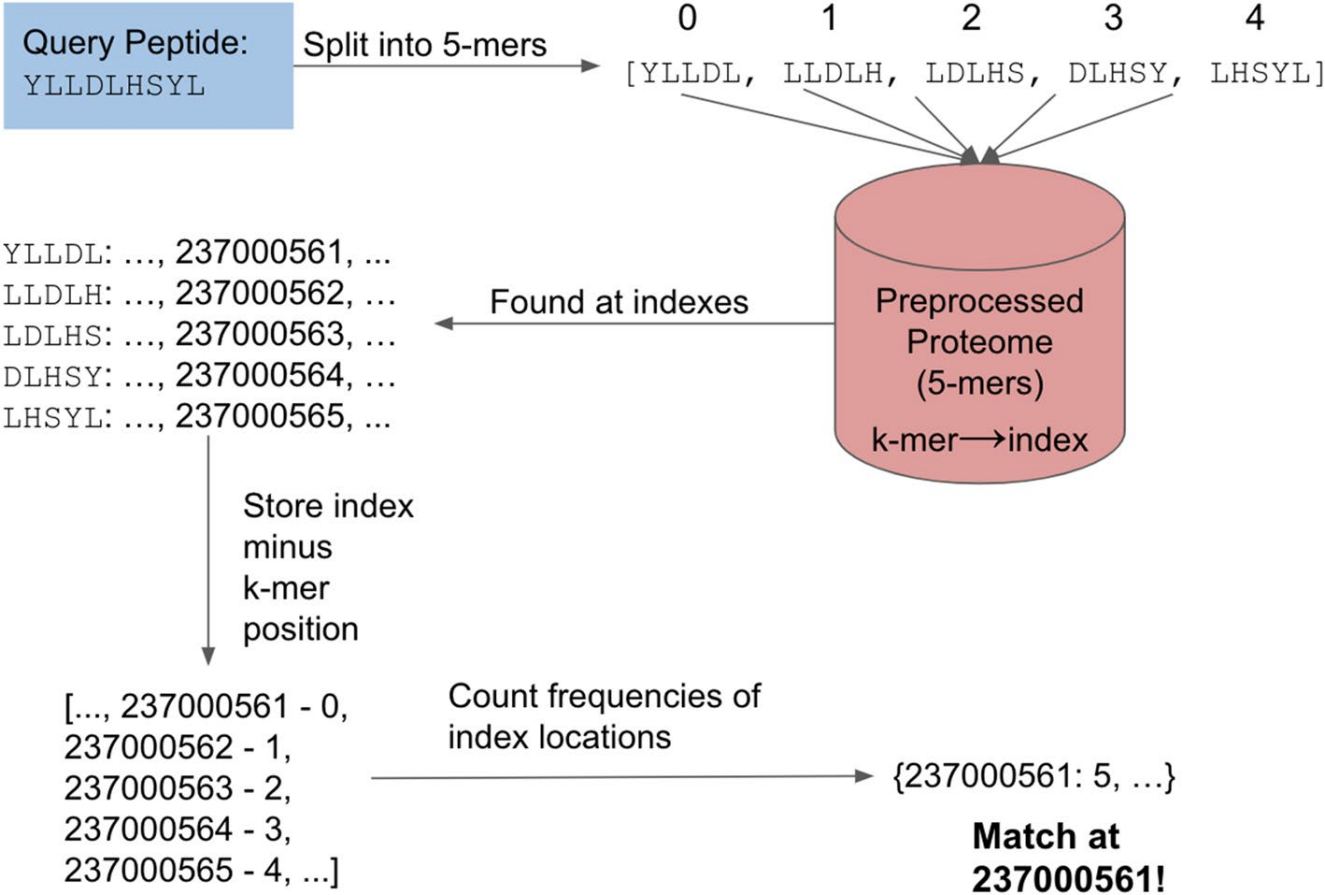
Exact matching search protocol. The query peptide is split into overlapping 5-mers since *k* = 5 for the preprocessed proteome in this example. Each 5-mer is searched in the preprocessed proteome using hash table lookups. The indexes where the 5-mers are found minus the position within the query peptide are recorded. Lastly, the frequencies of the index locations are counted, and if there are index counts equal to the number of 5-mers from the query peptide (five 5-mers in this example), then a match is found

For exact matching, each query peptide is broken up into the appropriate *k*-mers based on the preprocessing carried out on the reference proteome, and each *k*-mer is assigned a sequential index. For example, given a proteome preprocessed with *k* = 5, the peptide YLLDLHSYL can be split into overlapping 5-mers: (0) YLLDL, (1) LLDLH, (2) LDLHS, (3) DLHSY, (4) LHSYL (Fig. 1). For an exact match to be found, all of the *k*-mers must be found in the proteome in consecutive order. The PEPMatch algorithm checks that these conditions are met by subtracting each *k*-mer index (in the first example, 0 through 4) from the corresponding proteome index where the *k*-mer match was found (the example *k*-mers were found in indices 237,000,561 through 237,000,565) and recording the result. Thus, in the case of an exact match, the start index (in the example, 237,000,561) is recorded for each *k*-mer (5 times), which indicates that all *k*-mers are aligned consecutively. Alternatively, to maximize performance, this peptide can be split into two overlapping 5-mers: (0) YLLDL and (1) LHSYL, and only these two would need to be checked for consecutive alignment. We then use the start index location to map back to the protein within which the query peptide is found. Ideally, the optimal *k* would be the length of the peptide; however, since multiple peptides of different lengths can be searched and we want to minimize preprocessing, using a smaller *k* can cover more use cases without sacrificing much performance. If a given peptide contains multiple exact matches within a proteome, all are guaranteed to be found by PEPMatch.

#### Mismatch searching

Mismatching is done slightly differently but has similar steps involved. Given a query peptide and a number of allowed mismatches within it, we utilize the pigeonhole principle [19] to find the optimal *k* for the preprocessing step and use it to search for matches. The pigeonhole principle states that if *n* objects are put into *m* locations, where *n* < *m*, then at least one location must be empty. We apply this to sequence searching: given a number of mismatches, a peptide can be split up into *k*-mers such that, if a match exists, at least one *k*-mer would be exactly matched. This means we can vary either *k* or the number of mismatches to guarantee finding every match within the given proteome. The optimal *k* for a given peptide length, *l*, and the number of mismatches, *m*, is determined by the following equation:

1 $$k = \left[ {\frac{l}{m + 1}} \right]$$

We can also find the maximum number of mismatches given *k* and *l*:

2 $$m = \left[ {\frac{l}{k} - 1} \right]$$

Note that each equation takes the floor function if *k* or *m* is not an integer. Equation (1) will be used most often as the user is more likely to have a query peptide of a given length and would like to specify the mismatch allowance.

**Fig. 2:**
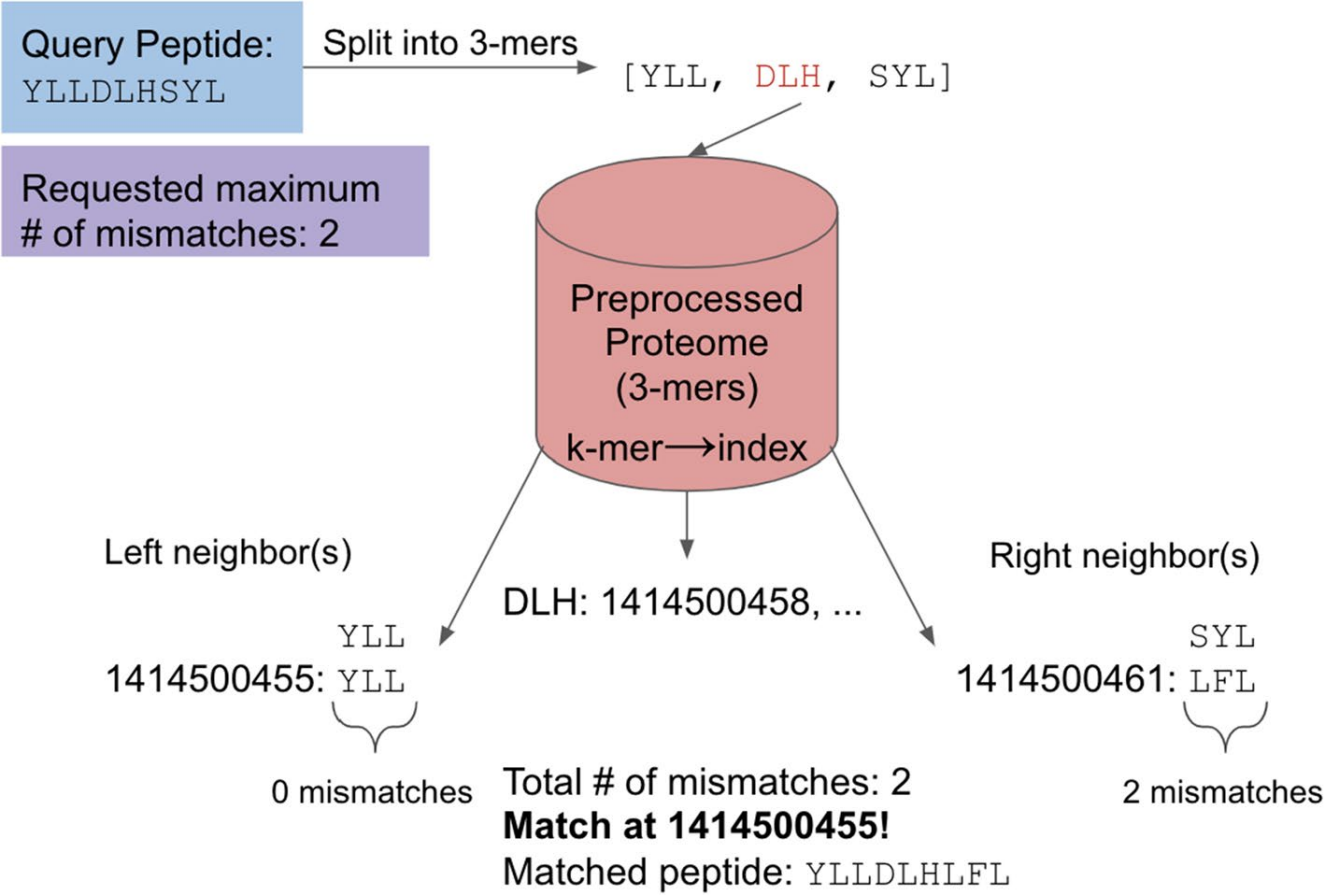
Mismatch search protocol. Given the query peptide of length 9 and the specified number of mismatches equal to 2, we determine that *k* needs to be 3, using Eq. (1). The peptide can also be split evenly since 9 is divisible by 3, so the *k*-mers are non-overlapping. The 3-mers are searched through the preprocessed proteome using hash table lookups. DLH is found at index 1,414,500,458, and the neighboring indexes are checked for Hamming distance. The left neighbor has 0 mismatches, and the right neighbor has 2 mismatches compared with the preprocessed proteome locations. In this case, the total number of mismatches is 2, which is equal to our threshold value, which means a match is found here

After *k* is determined, given the number of mismatches and length, we split the query peptide into the appropriate *k*-mers. The hash table lookups are performed within the preprocessed proteome in the same way as exact matching. Once a *k*-mer is found, the neighboring *k*-mers of the query peptide are compared to the neighboring *k*-mers within the preprocessed proteome. The Hamming distance of equal-length strings is the number of different letters at the same position (mismatches). We check these corresponding neighbors for their Hamming distance and combine the total number of mismatches for the query peptide. If there are less than or equal to the number of mismatches than the given allowance, it is a match (Fig. 2). We then record these corresponding *k*-mers and combine them to determine the matched sequence.

#### Best match searching

A valuable feature of peptide searching is finding the best match within a given proteome, defined as the match within a proteome with the lowest number of mismatches. Our solution for this problem is to perform the preprocessing step multiple times on a proteome for different *k* values. After this, we perform the exact match search once and mismatching search protocol multiple times using the different preprocessed data while increasing the mismatch threshold. If the query contains multiple peptides, those finding a match in earlier searches are removed from the next mismatching search until every query peptide has been matched.

**Fig. 3:**
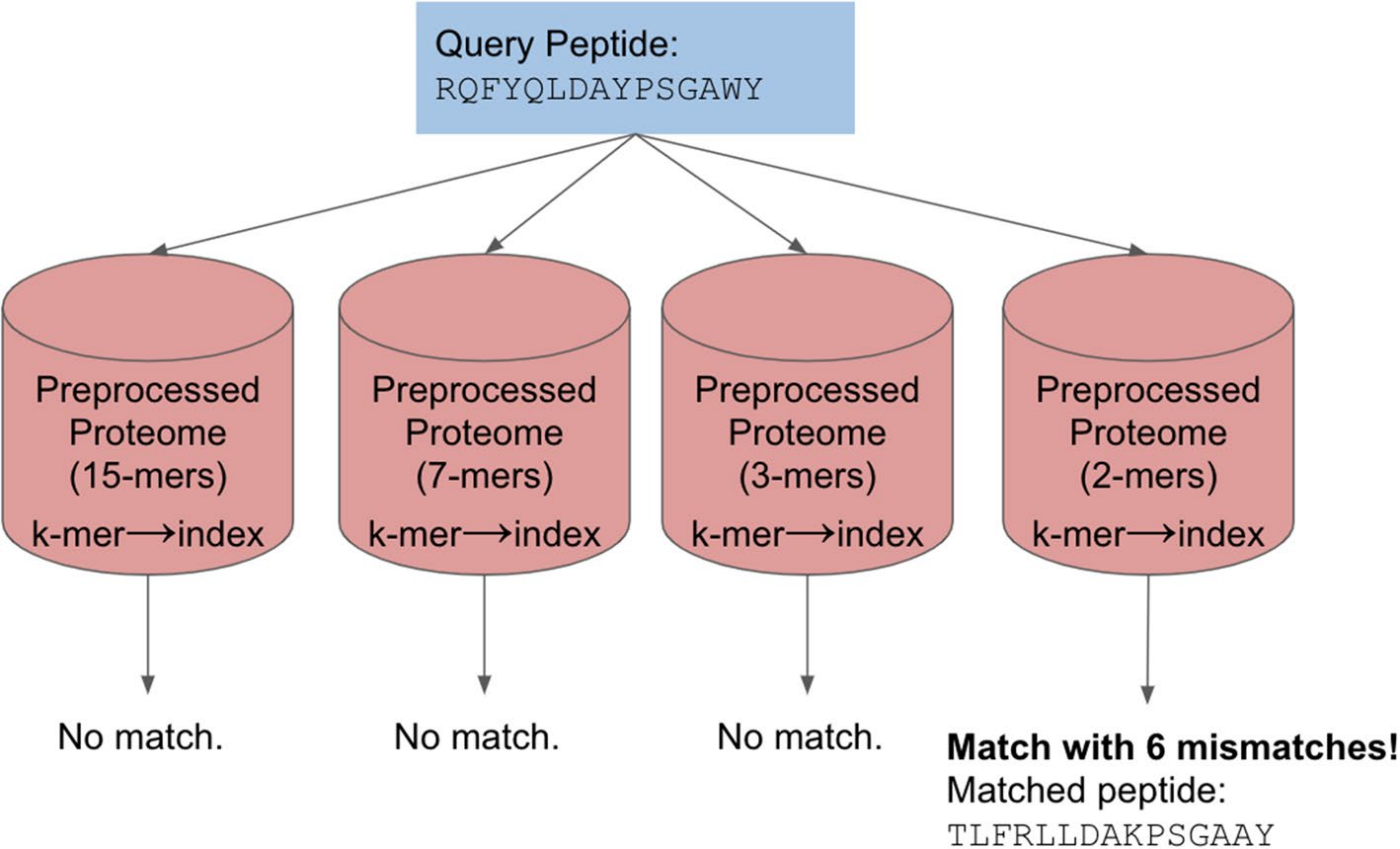
Best match searching feature. The proteome is preprocessed multiple times, starting with 15 (length of the query peptide) and halving *k* until we reach 2. We preprocessed the proteome four times using 15-mers, 7-mers, 3-mers, and 2-mers. The mismatching protocol uses the calculated maximum number of mismatches for each search. This is done until a match is found; we found a match with six mismatches in this case. If we had found a match in an earlier search, the subsequent searches would not have been performed

We first preprocess the proteome using *k* equal to the length of the query peptide (*l*), searching for exact matches only. Then, we preprocess using *k* = *l*/2, rounding down if *l*/2 is not an integer. We then continue halving and preprocessing until we reach *k* = 2. A preprocessed proteome of *k* = 1 is the same as brute force searching, so we do not go further. After all preprocessing is complete, we search the proteome starting with the highest *k* and work down until a match is found (Fig. 3). Using Eq. (2), we determine the maximum number of mismatches for the *k* value utilized and use that as our threshold for each search.

## Results

### Establishing benchmark

To compare the performance of different tools for peptide searching, we established benchmark datasets and metrics to evaluate the performance of different tools and algorithms. We developed a framework that provides a set of query peptides, a reference proteome, a mismatch frequency threshold, and a file of expected output. A tool that accepts these inputs can then be plugged into this framework to test itself against the methods available. The framework tracks the time it takes to preprocess the proteome, preprocess the query (if a tool performs such a task), perform the search, and then evaluate the output’s recall. In the evaluation of method performance, recall and search time are taken into consideration. Recall is defined as the percentage of actual positive matches the method correctly identifies. All the methods report the query peptide, the matched sequence, the match’s protein ID, and the match’s index position within the protein. We used this framework to run the datasets with the abovementioned methods and generated the results below.

Since building a framework to test these many methods was necessary, we have attempted to make it easier for users to test other possible methods with our codebase. The framework we created to establish these benchmarks can allow an individual to write a Python wrapper around their tool, which can be written in any language, and “plug” it into our benchmarking code. The wrapper must accept standardized inputs and give standardized outputs established by this benchmark. Instructions for testing a new tool can be found at https://github.com/IEDB/PEPMatch/tree/master/benchmarking.

### Benchmark results

#### Exact matching: MHC class I eluted ligands dataset

**Fig. 4:**
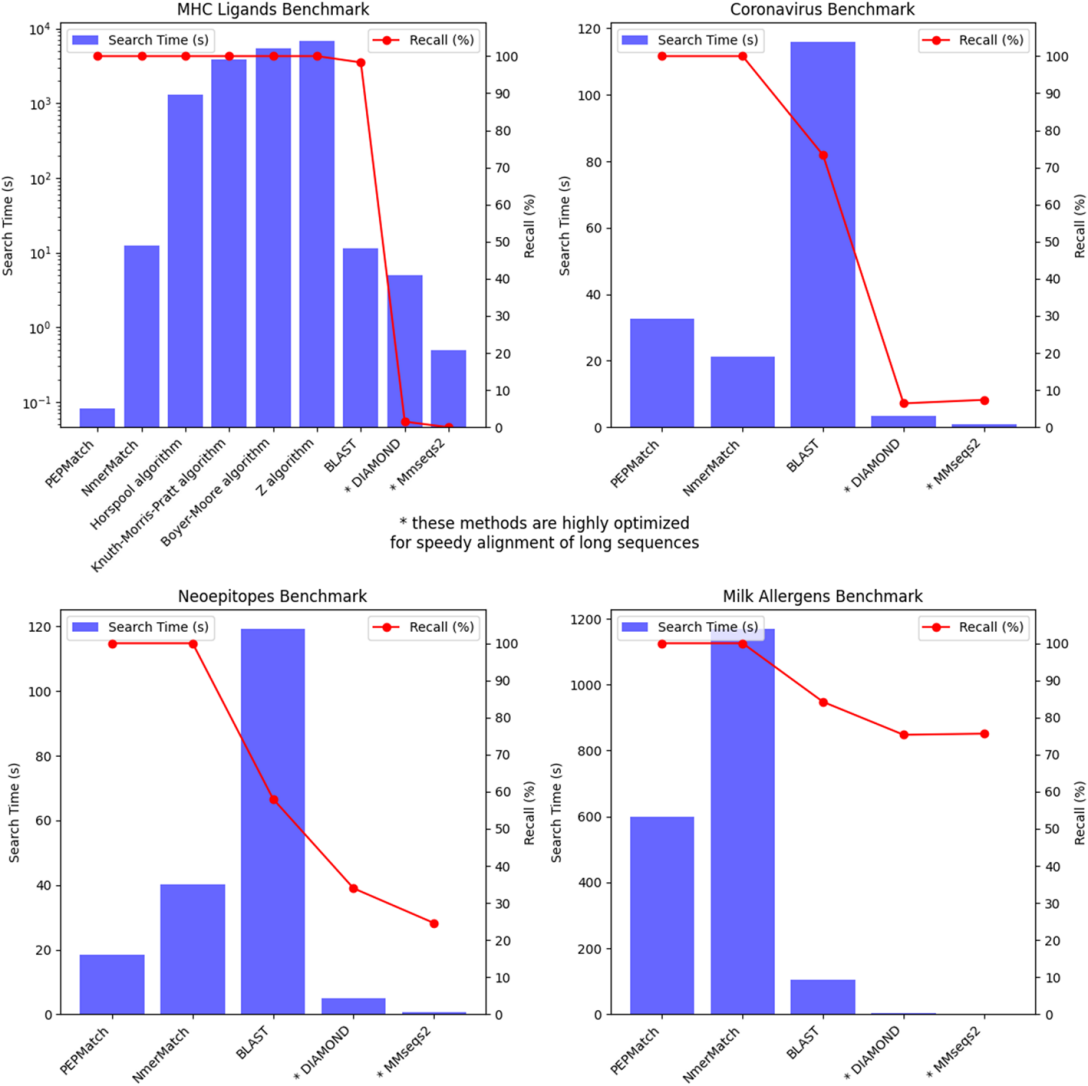
Search times and for the benchmarks. The methods tested for each benchmark with their time to complete their search (blue bars) and the recall as a percentage of matches found (red dots). The search times (in seconds) are shown on the left y-axis, with their respective accuracies on the right y-axis. The search time for the MHC ligands benchmark is on a logarithmic scale due to the major differences between the methods. PEPMatch is many orders of magnitude faster for the exact matching benchmark (MHC ligands) and can achieve 100% recall in finding matches for all the benchmarks. It is only outperformed in the coronavirus epitope benchmark by NmerMatch when factoring for recall

We compared the methods as explained above by the total time for the task, which is composed of the time it took to preprocess the proteome, preprocess the query, and search the peptides (Fig. 4). First, we tested the performance of all methods in finding exact matches in the human proteome for a dataset containing 2000 9-mers (Table 1). Proteome preprocessing was relevant for five methods: PEPMatch, NmerMatch, BLAST, DIAMOND, and MMseqs2. PEPMatch and NmerMatch used a significant fraction of the total time preprocessing the proteome (39.6 s and 53.7 s, respectively). BLAST, DIAMOND, and MMseqs2 preprocessed the proteome much quicker, taking between 0.25 and 2.65 s. The other exact matching methods do not perform any preprocessing step. Only one of the methods, NmerMatch, performed preprocessing on the query peptides, which took only 0.006 s. Search time varied from 0.08 s (PEPMatch) to 113 min (Z algorithm). Six out of the nine methods found every match with 100% recall. BLAST found 98.3% of all true matches, while DIAMOND and MMseqs2 found < 2% of the true matches. None of the methods matched the 1000 shuffled peptides anywhere in the human proteome. Overall, PEPMatch was the second slowest method for proteome preprocessing. However, it was several orders of magnitude faster in search time than the other methods. Total time was the shortest for MMseqs2, DIAMOND, and BLAST, followed by NmerMatch, PEPMatch, and the remaining string-searching algorithms.

**Table 1:** Results from the MHC class I dataset

Method	Proteome preprocessing time (s)	Query preprocessing time (s)	Searching time (s)	Total time (s)	Recall (%)
PEPMatch	39.6	N/A	0.08	39.7	100
NmerMatch	53.7	0.006	12.3	66.0	100
BLAST	1.27	N/A	11.3	12.6	98.3
DIAMOND	0.25	N/A	5.01	5.26	1.5
MMseqs2	2.65	N/A	0.50	3.15	0.0
Horspool	N/A	N/A	1,310	1,310	100
Boyer-Moore	N/A	N/A	5,424	5,424	100
KMP	N/A	N/A	3,807	3,807	100
Z	N/A	N/A	6,782	6,782	100

#### Mismatching: SARS-CoV-2 and neoepitope datasets

Next, we tested the performance of the methods that calculate mismatching (this excludes the string searching algorithms: Horspool, Boyer-Moore, KMP, and Z). We aimed to find all matches in the entire betacoronavirus genus for a SARS-CoV-2 dataset containing 628 peptides of varying lengths (8–15) up to and including 2 mismatches (Table 2). Proteome preprocessing time varied from 0.15 s (DIAMOND) to 214 s (NmerMatch). This step increased significantly for NmerMatch because it performs this step for each length of the query peptides. Query preprocessing is only done by NmerMatch, and this took 0.003 s. Search time varied from 0.77 s (MMSeqs2) to 115.9 s (BLAST). Recall was 100% for PEPMatch and NmerMatch but was considerably lower for the other methods. BLAST found 73.3% of the matches, whereas DIAMOND and MMSeqs2 only found 6.4% and 7.4%, respectively. The total time for the task was shortest for MMseqs2 and DIAMOND, followed by PEPMatch, BLAST, and then NmerMatch.

**Table 2:** Results from the SARS-CoV-2 dataset

Method	Proteome preprocessing time (s)	Query preprocessing time (s)	Searching time (s)	Total time (s)	Recall (%)
PEPMatch	34.1	N/A	32.6	66.7	100
NmerMatch	214	0.003	21.3	235.9	100
BLAST	0.51	N/A	115.9	116.4	73.3
DIAMOND	0.15	N/A	3.45	3.60	6.4
MMseqs2	1.83	N/A	0.77	2.60	7.4

We also tested the methods on a neoepitope dataset composed of 620 15-mers (Table 3). We wanted to find all matches in the human proteome with up to and including 3 mismatches. The time it took to preprocess the proteome varied from 0.24 s (DIAMOND) to 50.4 s (NmerMatch), and again, query preprocessing was only done by NmerMatch, which took 0.002 s. Search time was significantly faster for MMseqs2 and DIAMOND at 0.59 and 4.93 s, respectively, whereas BLAST took the longest at 119.2 s. PEPMatch took 18.4 s to search, and NmerMatch took 40.1 s. Recall was higher on this dataset for the alignment tools BLAST, DIAMOND, and MMseqs2 at 58.1%, 34.0%, and 24.6%, respectively. PEPMatch and NmerMatch were able to find all matches with 100% recall. Total time was longest for BLAST and shortest for MMseqs2.

**Table 3:** Results from the neoepitopes dataset

Method	Proteome preprocessing time (s)	Query preprocessing time (s)	Searching time (s)	Total time (s)	Recall (%)
PEPMatch	13.3	N/A	18.4	31.7	100
NmerMatch	50.4	0.002	40.1	90.5	100
BLAST	1.28	N/A	119.2	120.5	58.1
DIAMOND	0.24	N/A	4.93	5.17	34.0
MMseqs2	2.28	N/A	0.59	2.87	24.6

#### Best match: milk allergen dataset

Finally, we wanted to test the task of finding the best match within a proteome. Here, we ran the methods on a dataset of 677 15-mers from milk allergens to find the best match in the human proteome (Table 4). Only PEPMatch and NmerMatch have best match features; however, we also searched using BLAST, DIAMOND, and MMseqs2 to observe how they would perform. Preprocessing the proteome varied from 0.25 s (DIAMOND) to 203.7 s (NmerMatch), and search time varied from 0.48 s (MMseqs2) to 19.5 min (NmerMatch). Again, PEPMatch and NmerMatch found all the best matches at 100% recall, whereas BLAST, DIAMOND, and MMseqs2 found ~ 75 to 85% of all matches. Total time was shortest for MMseqs2 and longest for NmerMatch.

**Table 4:** Results from the milk peptides dataset

Method	Proteome preprocessing time (s)	Query preprocessing time (s)	Searching time (s)	Total time (s)	Recall (%)
PEPMatch	45.5	N/A	600.3	645.8	100
NmerMatch	203.7	0.005	1,168	1,372	100
BLAST	1.30	N/A	105.3	106.6	84.2
DIAMOND	0.25	N/A	5.24	5.49	75.3
MMseqs2	2.25	N/A	0.48	2.73	75.6

## Discussion

In this paper, we present a benchmark for finding short peptide sequences in large sets of proteins. Unbiased benchmarking is important when comparing any in silico methods since certain parameters can be tweaked to show an advantage when overall there may not be. This benchmark is intended to be unbiased by running all methods on the same machine, comparing their speed and recall without the concern of gaining an unfair advantage by parameter manipulation. We also present a new tool, PEPMatch, that performs well in these benchmark tasks.

Overall, we show that the tools utilizing hash table lookups (PEPMatch and NmerMatch) were able to perform speedy exact matching and mismatching searches with 100% recall. The common string-searching algorithms (Horspool, Boyer-Moore, KMP, and Z) used in many other string-searching applications are too slow for this task despite finding every match. The development of alignment tools over many decades, including BLAST, DIAMOND, and MMseqs2, was foundational for allowing search with residue substitutions, insertions, and deletions taken into account. These tools, while often faster for the task of finding matches with residue substitutions, their accuracies are lower when compared to deterministic algorithms such as PEPMatch for short sequences. This is likely because the alignment tools use k-mers for gapped and ungapped seeding and extending to find alignments of long sequences. Finding shorter sequences in large datasets is a different problem, and this is the conceptual advantage of PEPMatch. This is one possible explanation for the low recall of the alignment tools, as they are optimized for longer sequence alignments and are not easily amenable to account for the short peptide sequences. Since they also consider insertions and deletions when doing their alignments, and since we only tested for substitutions, they had more difficulty finding every match. This is supported by the fact that their accuracies were higher with the neoepitopes and milk allergen datasets, which were all 15-mers, as opposed to the SARS-CoV-2 dataset, which ranged from 8 to 15 residues. MMseqs2 and DIAMOND also had extremely low recall for the MHC class I dataset, all 9-mers. In addition, given that the benchmark code and method implementation is hosted on GitHub, it is easy to rerun and change parameters for potentially better results.

Preprocessing a proteome before searching dramatically increases the search time speed. If multiple runs are expected to be done subsequently with similar datasets, the preprocessing step only needs to be performed once, and the preprocessed data can be stored. This dramatically reduces the total time. For example, in the MHC class I dataset, PEPMatch searches all 2000 9-mers in 0.08 s, which is only 0.2% of the total time for the task. BLAST performs the fastest in total time for this dataset and has a high recall (98.3%), though its searching time was over 100 times slower than PEPMatch. It is also worth noting that we set up the benchmark to check for the matched sequence, protein ID, and index position of the match, which ensures that a false positive would be excluded, hence the use of the recall metric. Lastly, the alignment tools do not have a best match feature, whereas PEPMatch and NmerMatch do. For this reason, they can find every best match despite their search and total time being much longer.

**Fig. 5:**
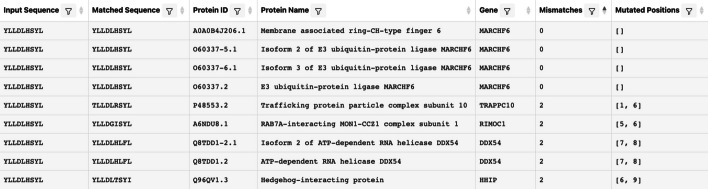
Example PEPMatch output from Next-Generation IEDB tools site. Using the peptide from the implementation description, searching up to 2 mismatches, this peptide maps to multiple proteins. The output includes the original sequence, the matched sequence, the UniProt protein ID, the protein name, the gene symbol, the number of mismatches, and the residue positions where those mismatches occur. The user has the option to also include the species or organism name for the proteome, the taxon ID for that organism, the start and end index positions within the protein that the peptide is found in, and the protein existence level, which is a value curated by UniProt providing the level of evidence which the protein exists

Progress must still be made towards speedier search times as proteomics involves big data, and researchers may need to perform searches on enormous datasets. For example, one may want to search within the entire bacteria domain, which contains > 164,000,000 proteins (queried on UniProt [20]). Speed improvements while maintaining 100% match recall are likely possible. The utilization of GPU programming and parallelization may significantly improve performance. Since peptide searching is a prevalent task in research, pipelines to and from PEPMatch can be established with other tools to facilitate the research process. In fact, on the IEDB Next Gen Tools site (https://nextgen-tools.iedb.org), PEPMatch is already piped to the IEDB peptide clustering and the MHC Class I prediction tools. Another potential extension for the tool might be ranking matches based on an amino acid substitution score. Certain amino acid substitutions are more frequent than others, which could be considered in the final output. It is also worth noting that peptides will often map to multiple proteins, especially when accounting for residue substitutions, which is important in immunology. Figure 5 shows an example output of the PEPMatch tool from the Next-Generation IEDB Tools website, which features a peptide that maps to multiple proteins.

## Conclusion

Our study introduces PEPMatch, a specialized tool for speedy and accurate short peptide sequence matching. Built on a k-mer mapping algorithm that preprocesses proteomes, the tool dramatically outpaces existing methods (such as BLAST) in speed without compromising recall. The study also presents an unbiased benchmarking framework that serves as a standard for evaluating future tools and methods for this task. The applications mentioned are only a few areas within immunology where such a tool could be utilized. This type of small sequence searching is vital for researchers working with T-cell epitopes, as described in the introduction. Ultimately, we believe PEPMatch, along with this benchmark, will help progress immunological research by providing highly accurate and speedy peptide searching.

### Supplementary Information

**Additional file 1:** 1000 MHC ligand peptides and 1000 shuffled peptides, all 9-mers, for the exact matching benchmark.

**Additional file 2:** 628 SARS-CoV-2 peptides of varying lengths for the 1st mismatching benchmark.

**Additional file 3:** 620 neoepitopes, all 15-mers, for the 2nd mismatching benchmark.

**Additional file 4:** 677 milk allergen peptides, all 15-mers, for the best match benchmark, annotated with their reactivity (True/False) from donor screening.

## Data Availability

Project name: PEPMatch. Project home page: https://github.com/IEDB/PEPMatch. Operating system(s): Platform independent. Programming language: Python. Other requirements: Python 3.7 + , pandas, NumPy, and Biopython. License: Non-Profit Open Software License 3.0 (NPOSL-3.0). Any restrictions to use by non-academics: None. All data and code for this work are available on the PEPMatch GitHub repository (https://github.com/IEDB/PEPMatch)

## Abbreviations

MHC: Major histocompatibility complex
IEDB: Immune epitope database
SARS-CoV-2: Severe acute respiratory syndrome coronavirus 2
KMP: Knuth–Morris–Pratt
HLA: Human leukocyte antigen
CEDAR: Cancer epitope database and analysis resource

## References

[1] Altschul SF, Gish W, Miller W, Myers EW, Lipman DJ (1990). Basic local alignment search tool. J Mol Biol.

[2] Edgar RC (2004). MUSCLE: a multiple sequence alignment method with reduced time and space complexity. BMC Bioinform.

[3] Buchfink B, Xie C, Huson DH (2015). Fast and sensitive protein alignment using diamond. Nat Methods.

[4] Steinegger M, Söding J (2017). MMseqs2 enables sensitive protein sequence searching for the analysis of massive data sets. Nat Biotechnol.

[5] Trolle T, McMurtrey CP, Sidney J, Bardet W, Osborn SC, Kaever T, Sette A, Hildebrand WH, Nielsen M, Peters B (1950). The length distribution of class I restricted T cell epitopes is determined by both peptide supply and MHC allele specific binding preference. J Immunol Baltim Md.

[6] Chang ST, Ghosh D, Kirschner DE, Linderman JJ (2006). Peptide length-based prediction of peptide-MHC class II binding. Bioinforma Oxf Engl.

[7] Vita R, Mahajan S, Overton JA, Dhanda SK, Martini S, Cantrell JR, Wheeler DK, Sette A, Peters B (2019). The immune epitope database (IEDB): 2018 update. Nucl Acids Res.

[8] Grifoni A, Weiskopf D, Ramirez SI, Mateus J, Dan JM, Moderbacher CR, Rawlings SA, Sutherland A, Premkumar L, Jadi RS (2020). Targets of T cell responses to SARS-CoV-2 coronavirus in humans with COVID-19 disease and unexposed individuals. Cell.

[9] Savage J, Johns CB (2015). Food allergy: epidemiology and natural history. Immunol Allergy Clin North Am.

[10] Cianferoni A, Muraro A (2012). Food-Induced Anaphylaxis. Immunol Allergy Clin North Am.

[11] Lewis SA, Sutherland A, Soldevila F (2023). Identification of cow milk epitopes to characterize and quantify disease-specific T cells in allergic children. J Allergy Clin Immunol.

[12] Sarkizova S, Klaeger S, Le PM, Li LW, Oliveira G, Keshishian H, Hartigan CR, Zhang W, Braun DA, Ligon KL (2020). A large peptidome dataset improves HLA class I epitope prediction across most of the human population. Nat Biotechnol.

[13] Boyer RS, Moore JS (1977). A fast string searching algorithm. Commun ACM.

[14] Horspool RN (1980). Practical fast searching in strings. Softw Pract Exp.

[15] Knuth DE, Morris JH, Pratt VR (1977). Fast pattern matching in strings. SIAM J Comput.

[16] Gusfield D (1997). Algorithms on strings, trees, and sequences: computer science and computational biology.

[17] O'Leary NA, Wright MW, Brister JR (2016). Reference sequence (RefSeq) database at NCBI: current status, taxonomic expansion, and functional annotation. Nucl Acids Res.

[18] Koşaloğlu-Yalçın Z, Blazeska N, Vita R (2023). The cancer epitope database and analysis resource (CEDAR). Nucl Acids Res.

[19] Trybulec, W.A. Pigeon Hole Principle. J Formaliz Math. 1990; 2: 0.

[20] The UniProt Consortium (2023). UniProt: the Universal Protein Knowledgebase in 2023. Nucl Acids Res.

